# Methods to optimize myxobacterial fermentations using off-gas analysis

**DOI:** 10.1186/1475-2859-11-59

**Published:** 2012-05-09

**Authors:** Stephan Hüttel, Rolf Müller

**Affiliations:** 1 Department of Pharmaceutical Biotechnology, Saarland University, Saarbruecken, Germany; 2 Helmholtz-Institute for Pharmaceutical Research Saarland, Helmholtz Centre for Infection Research and Pharmaceutical Biotechnology Saarland University, Saarbruecken, Germany

**Keywords:** Myxobacteria, Secondary metabolites, Carbon dioxide, Oxygen, Process automation, Production optimization, Bioprocess, OCQ, COQ, pO_2_, pCO_2_

## Abstract

**Background:**

The influence of carbon dioxide and oxygen on microbial secondary metabolite producers and the maintenance of these two parameters at optimal levels have been studied extensively. Nevertheless, most studies have focussed on their influence on specific product formation and condition optimization of established processes. Considerably less attention has been paid to the influence of reduced or elevated carbon dioxide and oxygen levels on the overall metabolite profiles of the investigated organisms. The synergistic action of both gases has garnered even less attention.

**Results:**

We show that the composition of the gas phase is highly important for the production of different metabolites and present a simple approach that enables the maintenance of defined concentrations of both O_2_ and CO_2_ during bioprocesses over broad concentration ranges with a minimal instrumental setup by using endogenously produced CO_2_. The metabolite profiles of a myxobacterium belonging to the genus *Chondromyces* grown under various concentrations of CO_2_ and O_2_ showed considerable differences. Production of two unknown, highly cytotoxic compounds and one antimicrobial substance was found to increase depending on the gas composition. In addition, the observation of CO_2_ and O_2_ in the exhaust gas allowed optimization and control of production processes.

**Conclusions:**

Myxobacteria are becoming increasingly important due to their potential for bioactive secondary metabolite production. Our studies show that the influence of different gas partial pressures should not be underestimated during screening processes for novel compounds and that our described method provides a simple tool to investigate this question.

## Background

The search for new antibiotics and other bioactive compounds has been intensified due to the development of multiple resistances in pathogenic bacteria and the lack of effective therapies against various - not only infectious-diseases [1,2]. Despite well-developed techniques to create synthetic compound libraries, the chemical variety and complexity found in natural molecules are often inaccessible via synthesis and lead structures from Nature are still in demand [3]. Bacteria are well-documented sources for these desired structures and, among microbes, myxobacteria are an important group, responsible for approximately 5% of the known bacterial compounds including molecules of considerable pharmaceutical interest [4,5]. Although thousands of different myxobacteria from diverse environmental sources have already been isolated and new species and even genera are still discovered [6], little is known about bioprocesses involving myxobacteria.

The myxobacterial genus *Chondromyces* appears to be a promising source for novel structures as several bioactive molecules, such as the chondramides and apicularens, have already been isolated and characterized [7,8]. To investigate the influence of the gaseous composition on myxobacterial growth and metabolic profile, SBCm007, a *Chondromyces* strain isolated in our laboratory, was chosen for its production of several uncharacterized compounds in addition to the known chondramides A–D. A second strain, SBCm002, which produces the apicularens A and B and two crocapeptins derivatives, both molecule classes showed activity against hepatitis C virus replication [9], was used to test if O_2_ and CO_2_ monitoring in the off-gas could help to optimize up- and downstream processing of myxobacterial fermentation processes.

The importance of a defined pO_2_ in the liquid phase to optimise production is documented by numerous examples of bioprocesses, mostly involving bacteria of the order *Actinomycetales*. In a *Streptomyces clavuligerus* culture, 100% pO_2_ saturation maintained over the whole cultivation time resulted in cephamycin C production 2.4-fold higher than that obtained from an uncontrolled experiment, where pO_2_ dropped below 40%. The authors showed that maintenance of 100% saturation was most critical during the exponential growth phase of the bacteria, where no production of cephamycin was observed. A drop from 100% to 50% after exponential growth delivered the same yields as 100% saturation over the whole cultivation time. Maintenance of 50% overall resulted in a four-fold lower yield, whereas biomass in all experiments was comparable [10]. In contrast, it was shown that high oxygen concentrations after the exponential growth phase of *Amycolatopsis orientalis* are crucial for vancomycin biosynthesis, although the biosynthetic machinery is present at high and low pO_2_[11]. These effects can be explained by regulatory influence of O_2_ on gene expression levels and enzyme function, but another important factor is the presence of O_2_ as a substrate, which can e.g. influence the proportion of tetracycline, oxytetracycline and chlortetracycline yield in several *Streptomyces* strains cultivated at elevated pressure [12]. A myxobacterial example is the heterologous expression of epothilone in *Myxococcus xanthus* under 50% and 0% pO_2_ saturation, which had a significant influence on the proportion of epothilones A–D in the overall yield. An additional product, Epo506, was observed preferentially at higher O_2_ concentrations [13].

**Figure 1:**
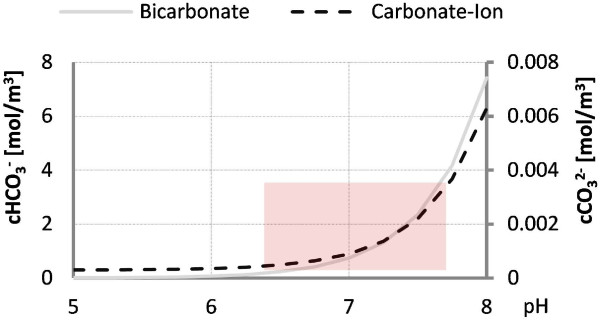
**Dependence of bicarbonate and carbonate ion concentration on pH.** Graph shows the calculated molar ratio of both substances at 1% CO_2_ saturation of the gas phase. The dissolved concentrations of CO_2_ and H_2_CO_3_ are dependent only on the concentration in the gas phase and not on the pH.

The evolution of CO_2_ under aerobic growth of microorganisms has been studied extensively and one major difference to O_2_ is that CO_2_ exists in the liquid phase as dissolved CO_2_ and carbonic acid (H_2_CO_3_) in equilibrium with the CO_2_ in the gas phase and carbonic acid converts, dependent on the pH of the solute into bicarbonate (HCO_3_^−^) and the carbonate ion (CO_3_^2−^), as shown in Figure 1. This complicates the investigation of the regulatory functions of CO_2_ itself because it is difficult to estimate which species is responsible for an effect. Particularly around pH 7, where myxobacterial fermentation usually occurs, an increase of both pH dependant species is observed. Possible effects on cells, enzymes, and product formation are reviewed extensively in [13,14] and, though it seems that higher concentrations correlate mostly with inhibiting effects under aerobic growth, there is evidence that low concentrations are at least somewhat beneficial and may be required for growth and production. In contrast, there have been reports that production of the antibacterial compound amylovorin L by *Lactobacillus amylovorus* is optimal under anaerobic conditions and high CO_2_ tensions. Similar findings are valid for induction of the toxic shock syndrome toxin 1 in *Staphylococcus aureus* MN8, where CO_2_ concentrations of about 7% and O_2_ concentrations of about 21% in the gas phase were required for optimal production.

Taken together, it becomes evident that, although much is known about O_2_ requirements, biosynthetic routes, and inhibitory or stimulatory effects of CO_2_, it is impossible to predict the influence of reduced or increased concentrations of these two gases on secondary metabolite production, particularly of new and unknown metabolites. Furthermore, the lack of literature where the effects of both parameters are observed in concert shows that there is an enormous potential for process optimization. In this study, we tested the influence of gas composition on the secondary metabolite profile of strain SBCm007. Our findings indicated the importance of a controlled gaseous environment. To address this issue, we developed a technique which allows, with minimal instrumental setup, the maintenance of both gases at defined concentrations during cultivation by using off-gas analysis and dissolved O_2_ as control parameters. Our studies further reveal that the introduction of two new parameters COQ and OCQ - both based on off-gas analysis-gives us a very sensitive tool that may be useful in process monitoring and automation. We demonstrate their use in condition optimization and the control of repeated batch cultivation with respect to better production and simplified downstream processing with promising results.

## Results and discussion

### Influence of O_2_ and CO_2_ tension on secondary metabolite production

This test was performed with a novel *Chondromyces* strain SBCm007 isolated in our laboratory. Due to unavoidable concentration differences in autogenously produced CO_2_ resulting from problems with the typical coagulative growth and thereby associated irreproducibility of inoculum density, a repeated batch setup was used when repetitive pattern state conditions were reached, as described in Materials and Methods. Thereby, the same culture could be used for all tests. The conditions for the tests were chosen from preliminary trial runs carried out between three to ten times which showed an effect on secondary metabolite production. The tests were performed in the order listed in Table 1, along with standard conditions before and after the test cultivations to ensure that no bigger deviations occur caused by the changed conditions or adaptive mutations. To rule out influences caused by shearing stress, the stirring rate was kept constant and pO_2_ maintained by changing the flow rate.

**Table 1:** **List of different test runs, the according gaseous composition and the method used to maintain pO**_2_*

**Low O**_**2**_ **Moderate CO**_**2**_	**Low O**_**2**_ **No CO**_**2**_	**Moderate O**_**2**_ **Moderate CO**_**2**_	**High O**_**2**_ **High CO**_**2**_	**High O**_**2**_ **No CO**_**2**_	**Moderate O**_**2**_ **High CO**_**2**_
pO_2_ 5%,	pO_2_ 5%,	pO_2_ 20%,	pO_2_ 55%,	pO_2_ 55%,	pO_2_ 20%,
CO_2_ 0.3–0.6%	CO_2_ < 0.1%	CO_2_ 0.2–0.3%	CO_2_ 1.0–2.0%	CO_2_ < 0.1%	CO_2_ 1.8%
Shearing stress comparable to standard conditions	Shearing stress comparable to standard conditions	Standard conditions	Shearing stress comparable to standard conditions	Shearing stress comparable to standard conditions	New Method: Shearing stress dependent on stirrer speed
Gaseous composition and pO_2_ regulated by aeration with ambient air	Gaseous composition and pO_2_ regulated by aeration with ambient air stripping of CO_2_ with nitrogen gas	Gaseous composition and pO2 regulated by aeration with ambient air	Gaseous composition and pO_2_ regulated by aeration with pure O_2_	Gaseous composition and pO_2_ regulated by aeration with ambient air	Gaseous composition and pO2 regulated by aeration with ambient air

*Trials were carried out in the presented order. Before and after the tests one standard run was carried out to ensure that no significant deviations are caused by the test runs.

**Figure 2:**
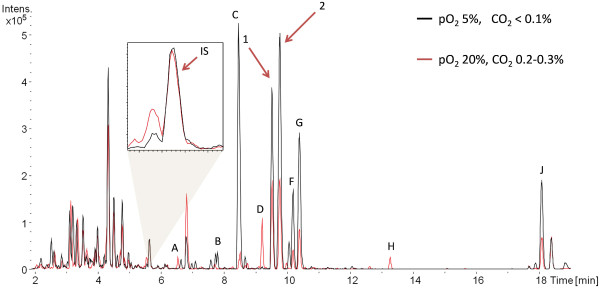
**Overlay of MS chromatograms of two extracts from a *Chondromyces *strain.** Overlay of two base peak chromatograms measured in positive ionisation mode. Peaks corresponding to a compound mentioned in the text are marked by the corresponding letter. 1 indicates chondramide A and 2, chondramide B. The small window enlarges the overlay of the peak corresponding to the internal standard (IS).

**Figure 3:**
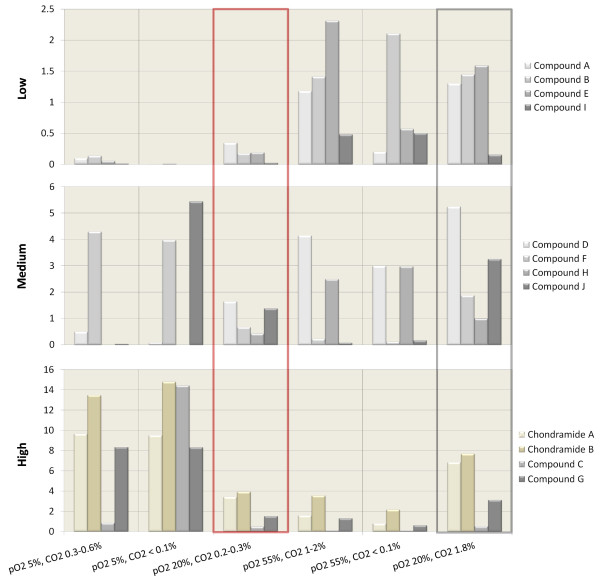
**Comparisons of the relative differences in productivity caused by different compositions of the gas phase inside the bioreactor.** The values are dimensionless as they are calculated based on relative peak area against an internal standard. Twelve target compounds are grouped into three major groups according to their relative peak areas. These results clearly show that generally accepted standard conditions, marked with a red box (pO_2_ 20%, CO_2_ 0.2–0.3%), are far away from optimal for any of the investigated compounds. The grey box indicates yields obtained with the method described in this study.

To compare the productivity under different conditions, the production of twelve target compounds (TC), including the highly cytotoxic chondramides A and B, was examined. These TCs were chosen when the preliminary tests showed reproducible influences caused by changes in gaseous composition. Figure 2 shows an example of an overlay of two base peak chromatograms measured in positive mode which were used for yield calculation. The twelve TCs are labelled A to J according to their retention time and marked when present in the extract. The overall productivity was calculated as described in Materials and Methods using sulfadimethoxine as an internal standard (see Figure 2) and the results are shown in Figure 3 as the relative productivity in comparison to the other conditions. The twelve compounds are characterized as low, medium, or highly abundant to enhance the clarity of the graph. The grey shaded box indicates yields obtained with a new method discussed in the next section and will not be considered here. The red shaded box marks the ‘standard’ conditions. The calculations clearly show the best production conditions for chondramides A and B are at low oxygen tension, as with compounds F and G. Production of compounds C and J seem to profit from low O_2_ and CO_2_ tension. Compounds A and E are best produced under high O_2_ and CO_2_ tension, whereas production of B, D, H and I seem to require only high O_2_ concentrations. Interestingly, the ‘standard’ conditions are suboptimal for production of all of the TCs.

Preliminary tests show highly cytotoxic activity for compounds F and G (IC_50_ about 28.6 nM and 44.2 nM on HCT-116 cells, data not shown) and weak cytotoxic and antibacterial activity in the micro-molar range for compound H (data not shown). For compound I, the increase in productivity under the tested conditions is not very high as shown in Figure 3. However, even a 20% improvement of overall yield in the low abundance group can uncover undetected activity or enable the production of sufficient amounts for chemical characterisation of a substance. For some compounds, very specific conditions seem to be required, as for C, E and J, which excludes the possibility for a single condition where all compounds can be produced in sufficient amounts. Furthermore, the application of standard cultivations usually only includes the control of pO_2_ and neglects CO_2_ concentration. Using the standard methods applied here, it was impossible to establish 100% reproducible conditions and the need for a method to maintain both gases at defined concentrations is obvious.

### Development of a method for maintenance of O_2_ and CO_2_ tension

**Figure 4:**
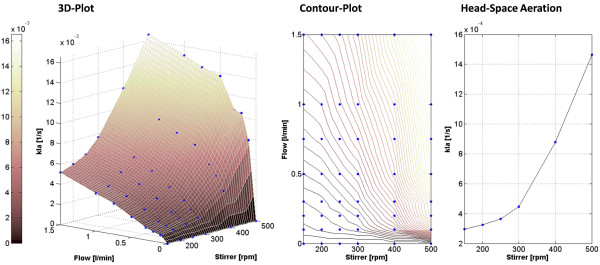
**3D and contour plots of k**_**l**_**a values with respect to stirrer speed and flow rate and k**_**l**_**a values obtained by headspace aeration with respect to stirrer speed.** Blue dots in all plots indicate measured values. The impact of the stirring rate is much higher on k_l_a then the influence of the flow-rate and. Higher flow rates result in a linear increase in k_l_a whereas higher stirring rates increase k_l_a exponential as illustrated in the 3D plot. High stirring rates and low flowrates deliver high k_l_a values. A further increase of the flow rate has just a minor effect on k_l_a as indicated in the contour plot. Low stirring rates have a minor effect when only headspace aeration is applied, while higher stirring rates cause an increase of k_l_a which can be at 500 rpm around 10% of that achieved by bottom sparging.

The influence of gas phase composition on the secondary metabolite profile shows the importance of maintenance of both gases inside the bioreactor. The lack of simple methods to achieve this led to the investigation of the dependency of k_l_a on stirring speed and flow rate. Figure 4 shows a 3D plot of k_l_a values with respect to these two parameters as measured in a bioreactor with water filling without any microorganism. It is obvious that, at low stirring rates, the increase in k_l_a at elevated flow rates has a linear dependency. At higher stirring rates, a sharp increase from zero flow to moderate flow rates occurs, followed by a linear increase. This effect can be explained by the following equations, which are generally used to calculate the oxygen transfer rate (OTR [mg/l*s]):

(1) $$OTR=k_{l}a\;\left(C_{O_{2}}^{*}-C_{l}\right)$$

where C_O2_^*^ [mg/l] is the maximum dissolved concentration of O_2_ at the actual conditions according to Henry’s law and C_l_ [mg/l] is the actual concentration of O_2_ dissolved in the liquid. k_l_a [1/s] is the mass or oxygen transfer coefficient per unit volume and can be expressed as:

(2) $$k_{l}a=k_{l}\;\frac{A}{V}$$

where k_l_ [m/s] is the mass transfer coefficient, A [m^2^] is the interface area between gas and liquid phase in the bioreactor and V [m^3^] is the liquid volume. This shows that there are two driving forces for mass transfer: the concentration difference (see Eq. 1) and the k_l_a value and its associated gas–liquid interface. Increasing k_l_a means increasing the interface area between liquid and gas phase (see Eq. 2). The latter can be achieved by increasing the flow rate, which results in a higher number of bubbles with approximately the same diameter and surface area, resulting in the observed linear increase of k_l_a with increasing flow rates. A higher mass transfer rate in common bioreactors can also be achieved by increasing the stirring rate, leading to greater dispersion of the bubbles and thereby to an exponential increase of bubble surface area. This explains the exponential increase of k_l_a in the 3D plot of Figure 4 when the stirring rate is increased.

Furthermore, the influence of O_2_ in the headspace volume on k_l_a with respect to stirring speed was investigated. To examine this, the headspace volume was exchanged permanently and no air was sparged through the liquid phase. Figure 4 shows that the effect is relatively low at 150 rpm but the transfer rates obtained through headspace exchange at 500 rpm were about 10% of the values obtained by sparging air through the liquid phase. This is due to greater dispersion of air from the surface to the liquid phase at higher stirring rates. The contour plot in Figure 4 illustrates that similar k_l_a values can be reached with a flow of 1.5 l/min and 150 rpm stirring rate or 0.1 l/min and 500 rpm, which can be partially traced back to headspace effects.

These combined characteristics of sparged air and head space effects can be used to control accumulation of CO_2_ in the bioreactor by setting pO_2_ under the control of flow rate and CO_2_ concentration in the off-gas under the control of the stirring rate. In both cases, a negative control deviation should lead to upregulation of the actuating variable and a positive deviation, to downregulation (The setting of the underlying PI control is listed in the Methods section). A CO_2_ concentration below the set point, for example, would lead to an increase in stirring rate, thereby increasing k_l_a and reducing the required flow rate. A decreased flow rate results in a reduced exchange of the headspace volume, thereby allowing CO_2_ accumulation until the set point is reached and the actuating variables remain constant. pCO_2_ in the liquid phase is assumed to be in equilibrium to the gas phase CO_2_ according to Henry’s law in an ideally mixed tank because the limiting step in gas exchange is the diffusion of CO_2_ in the liquid phase. Results from literature confirm this, at least for non-viscous culture broths where gas diffusion is not hindered [15,16].

In this study *Saccharomyces cerevisiae* was used for all initial trials because test runs could be performed after 1–2 days whereas preparation of myxobacterial starter cultures took usually between five to seven weeks. The underlying principles of the effects relevant for this study could be investigated regardless whether the respiratory active organism was of myxobacterial or fungal origin, results could be transferred to myxobacterial cultivations. Our initial testing showed that the influence of shear stress on the microbes must be considered because increased CO_2_ levels can only be achieved through higher stirring rates. A test cultivation with our model organism revealed that CO_2_ levels between 0.5–8.0% can be achieved with stirring rates between 200–800 rpm in minimal media with endogenously produced CO_2_ and ambient air when pO_2_ is set to 20% (data not shown). If higher CO_2_ concentrations are required, the use of pure O_2_ gas can reduce the required flow rate further and increase accumulation of CO_2_. To reduce the initial loading phase when endogenously produced CO_2_ is used an external CO_2_ loading pulse is conceivable. For very low CO_2_ concentrations at low pO_2_, the additional stripping of CO_2_ with nitrogen gas is required (see Figure 3, compounds C and J). Shang and co-workers presented ‘the autogenous CO_2_ method’ where they increased CO_2_ concentration by exchange of air gassing with pure oxygen and reducing thereby the flow rate [17]. This method allows only variations of CO_2_ but no real control. Using the proposed method allows the operator a tight control and also defined changes and growth phase dependent adaption of CO_2_ concentration during fermentations (data not shown).

**Figure 5:**
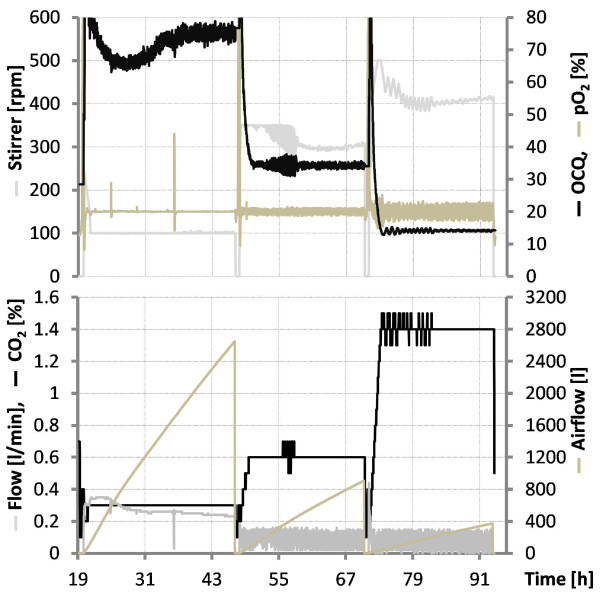
**Fermentation graphs of a repeated batch cultivations of *Chondromyces *strain SBCm007.** CO_2_ was kept at 0.3, 0.6 and 1.4% and pO_2_ at 20%**.** Both concentrations were maintained with the method described in this study. The steps in CO_2_ concentration indicate medium exchanges. The graph illustrates the calculated value OCQ, pO2, stirrer speed, flow, concentration of CO_2_ in the gas phase, and airflow (overall amount of air pumped through the bioreactor). The reduced flow rate at higher CO_2_ values led to ‘flow pulses’ because flow rate dropped below 0.2 l/min, the limit of the mass-flow controller which did not affect functionality. The belly of the OCQ value in the first batch, which was expected to be constant, is due to a lower operational limit of the stirrer and illustrates the sensitivity of the calculated OCQ value because deviations in the CO_2_ concentration caused by this are not yet obvious but already indicated by the OCQ.

Figure 5 shows a fermentation graph of a repeated batch cultivation performed in triplicate with strain SBCm007. pO_2_ was set at 20% in all cases and CO_2_ concentration at 0.3, 0.6 and 1.4%. The results clearly demonstrate the applicability of this technique. The last column in Figure 3 shows yields obtained with that method from a cultivation of SBCm007 at 20% pO_2_ saturation and 1.8% CO_2_ concentration in the off-gas compared with results of the above section. For compounds A, B and D, production is best under these conditions. For all compounds, production is superior to standard conditions indicating the potential for optimisation.

### OCQ and COQ as new parameters for process automation

As shown in the previous section, the gas composition of the off-gas is a direct result of respiratory activity, flow rate, and power entry into the media. A closer look at the influence of CO_2_ and O_2_ revealed that both gases deliver very specific growth phase- and nutrient-dependent curves which can be measured via off-gas analysis based on infrared measurement for CO_2_ and zirconium dioxide membrane potential for O_2_. Calculated values like OUR, carbon dioxide evolution rate (CER), and respiratory quotient RQ were not sufficiently sensitive and too susceptible to perturbations for use as monitoring or automation signals due to the weak respiratory activity of the investigated organisms. This leads to relatively small differences of CO_2_ and O_2_ concentrations in comparison to fresh air. This fact makes calculation of useful CER/OUR values impossible due to the experimentally observed “noise” (data not shown). Therefore other possible readouts were investigated.

**Figure 6:**
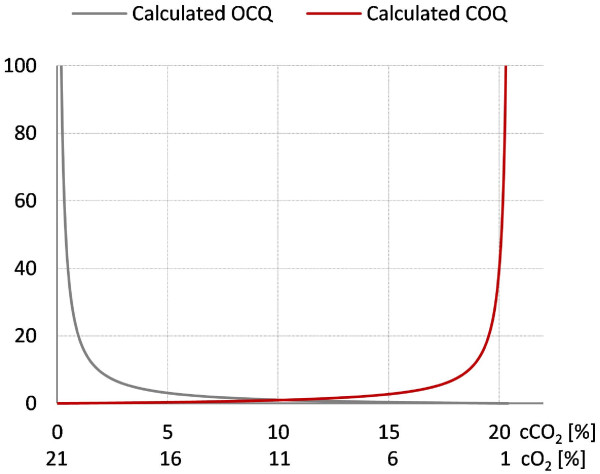
**Calculated OCQ and COQ curves.** The graph shows the calculated OCQ (cO_2_/cCO_2_) and COQ (cCO_2_/cO_2_) values according to the corresponding O_2_ and CO_2_ concentration in the off gas. Assumed is a RQ of 1 meaning for every respired portion of O_2_ a equivalent portion of CO_2_ is produced. The curve progression indicates best sensitivity for OCQ at concentrations of O_2_ and CO_2_ on the left side of the graph which usually occur at low respiratory activity.

**Figure 7:**
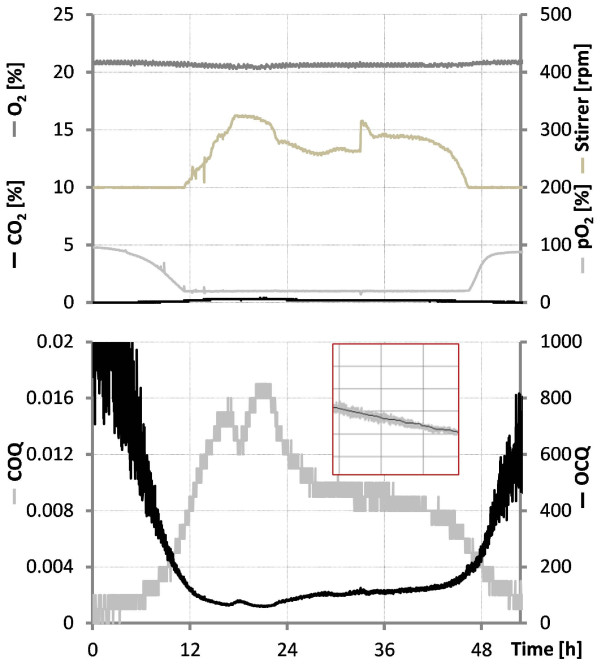
**Fermentation graphs of a *Saccharomyces cerevisiae *cultivation.** The graph shows O_2,_ CO_2_, pO_2_ and stirrer speed of a process where pO_2_ was controlled via agitation. Also shown are the calculated parameters COQ and OCQ. The curves obtained are highly reproducible and indicate differences in the respiratory activity of the fermented organism. The red shaded box shows OCQ (grey) and averaged OCQ (black) above 3 min which drastically reduces the noise from measurements.

**Figure 8:**
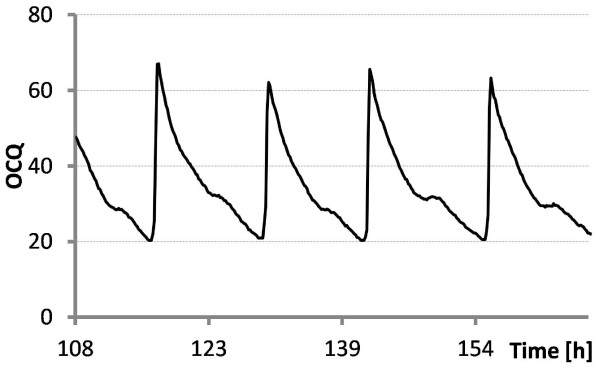
**OCQ of a repeated batch cultivation of *Chondromyces *strain SBCm002.** The pO_2_ was maintained via aeration and the stirring rate was kept constant, resulting in an increase of CO_2_ and decrease of O_2_ in the off-gas once respiratory activity decreased. The resulting OCQ curves are highly specific and a lower threshold of this value was used to trigger automated media exchange and metabolite capturing. The culture was maintained for 22 days with approximately 1.5 media replacements daily.

We found that division of off-gas O_2_ by off-gas CO_2_ concentration (O_2_-CO_2_-Quotient or OCQ) and vice versa (COQ) might be valuable tools for these purposes. The fact that O_2_ and CO_2_ concentration in the off-gas are inversely related with respect to biological activity ‘amplifies’ the calculated signals. This means that higher biological activity reduces O_2_ and increases CO_2_ in the off gas resulting in a calculated parameter where influences of both gases are combined in one value increasing sensitivity in comparison to OUR and CER where only one factor is used for calculation. Further amplification comes from the effects described in the above section. For example maintenance via flow rate leads to high OCQ values at high biological activity because CO_2_ is constantly blown out and mass transfer of O_2_ from air into media is not comparable to mass transfer achieved with increasing stirring rates leading to high O_2_ concentrations in the off gas. Vice versa at low respiratory activity OCQ reaches low values because the flow rate decreases and the mass transfer from the headspace becomes more important leading to accumulation of CO_2_ and reduction of O_2_ in the off gas. The resulting OCQ curve in a batch process with pO_2_ maintenance via flow rate starts usually at low values, increases when the cells reach the exponential growth phase and drops again when the respiratory activity decreases due to nutrient depletion. The corresponding COQ curve behaves inversely as it represents the reciprocal value of OCQ. The shape of these curves can be influenced by the initial setting of the stirring rate and must be tested beforehand. When pO_2_ is maintained via stirring rate the resulting OCQ curve starts at high values due to the constant blowing out of CO_2_ at the beginning of the cultivation where only low respiratory activity takes place. Once cells reach the exponential growth phase CO_2_ is produced and O_2_ concentration in the exhaust gas reduced by mass transfer from air into liquid phase resulting in a drop of the OCQ value. At the end of the cultivation OCQ rises again. The overall shape here is dependent on the initial flow rate and must also be tested beforehand. Figure 6 shows the calculated dependency of OCQ and COQ when ambient air is used as O_2_ source and the cultivated organism has a RQ of 1 meaning production of one portion CO_2_ removes the same portion of O_2_ from the exhaust gas. This calculation indicates best sensitivity of OCQ at lower CO_2_ concentrations and vice versa best sensitivity at higher CO_2_ concentrations for COQ. The setting of the stirring and flow rate allows moving conditions either to the right or the left side. This finding makes especially OCQ a valuable tool for respiratory weakly active organisms like myxobacteria. After averaging the signals, the measurement noise is filtered out and relatively smooth signals are obtained. Figure 7 illustrates the time-dependent development of both parameters during a batch cultivation of our test organism *Saccharomyces cerevisiae* were pO_2_ is maintained via stirring rate. A direct comparison of these parameters and CER/OUR is not possible since the latter ones are independent from cultivation conditions which is not the case for OCQ/COQ. Both parameters are dependent on the applied conditions and react very sensitive in their specific range on changes of stirring speed, flow rate and respiratory activity. The curves obtained are typical and highly reproducible for cultivations performed under the same conditions. The reproducibility of these curves was used to automate a repeated batch cultivation of *Chondromyces* strain SBCm002 once the culture reached repeated pattern state. The pO_2_ in this cultivation was maintained via flow rate. The resulting OCQ curve (see. Figure 8) is slightly different from those described earlier in the text as typical for this type of pO_2_ maintenance. This is due to the relative high amount of biomass at the beginning of a cycle in comparison to usual batch cultivations. This leads to a skipping of the lag phase resulting in a steep increase of OCQ after media exchange and then a slow decrease when nutrients are depleted. The time of media exchange was set under control of a lower threshold of OCQ which was the actual value when the time point of manual media exchange based on experience of the experimentator was reached. Then the cultivation was carried out under full automation using the control software of the bioreactor with approximately 1.5 media exchanges per day and subsequent capturing of metabolites on an adsorber resin (Amberlite XAD16) until the process was stopped after 22 days. Figure 8 shows an excerpt of the OCQ curve, where a lower threshold was used to trigger media exchange.

**Figure 9:**
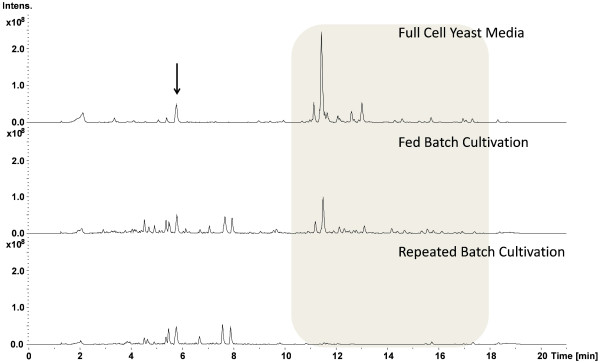
**Base peak chromatograms of extracts from immediate or sequential metabolite capture.** Extracts from a media blank, batch cultivation with immediate addition of XAD and batch cultivation with subsequent capturing of metabolites were measured in positive ionisation mode. The chromatogram of the media blank shows substances which are able to bind to the resin and prevent thereby binding of products and complicate further downstream processing. The batch cultivation where XAD was included illustrates that these substances are still present after the cultivation and can be only reduced when XAD16 is added at a very late time point when the media is already depleted as done in the repeated batch cultivation. The black arrow indicates the peak of the internal standard used for subsequent quantification.

**Figure 10:**
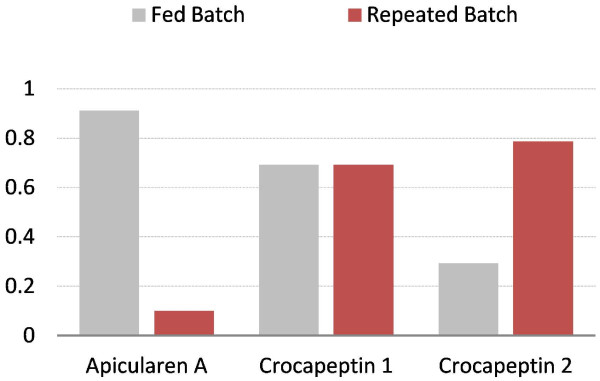
**Comparison of the relative productivity of two substance groups produced in a repeated batch with subsequent capturing and batch with direct capture cultivation.** The graphs represent relative peak areas in comparison to sulfadimethoxine as internal standard. For apicularen A, batch cultures are clearly superior. For crocapeptins results are comparable for one derivative and better for another derivative when metabolites are captured subsequently.

One advantage of this method is that it required just one cultivation cycle to use the experience of the microbiologist and incorporate it into an automation script running on the control system of the bioreactor by simply using a lower threshold of the OCQ value to trigger the media exchange. By using this very simple automation procedure it was possible to use the potential of the producing cells for more than 20 days before the process was stopped manually. Batch processes in comparison are usually finished in less then one week and fed batch processes in less then two weeks due to self intoxication of the cells as a side effect of the complex cultivation medium. To compare the productivity of this process with a common batch cultivation the supernatant of one cultivation cycle was captured separately and matched with a comparable batch process with cultivation in presence of the adsorber resin. XAD16 is usually added directly to the culture to prevent degradation of secondary metabolites during the cultivation by adsorbing them to the resin. This comparison revealed that the undesired binding of media compounds to the resin is drastically reduced in repeated batch mode. This so called resin fouling is usually a major problem in downstream processing of material produced with conventional cultivation techniques due to reduced binding capacity of the resin and purification problems. Figure 9 shows three base peak chromatograms measured in positive ionisation mode of a medium extract, an extract of the batch cultivation with direct addition of XAD and an extract of a single cycle of the repeated batch with subsequent capturing. The reduced binding of media compounds is shown with the shaded box. Figure 10 shows a comparison of the overall productivity of apicularen A and two crocapeptin derivatives from these cultivations. Both substance classes attracted attention in an anti HCV screening [9]. The yield of apicularen A is reduced in the repeated batch cultivation whereas the yields of the crocapeptins are comparable or even better than with addition of XAD, a phenomenon observed for several other compounds also (data not shown). This indicates a drawback of the method when degradation sensitive compounds are targeted but can be of advantage when production of metabolites is upregulated when these molecules are not captured directly.

These findings indicate that production must be optimized for every single compound, but in cases where compounds are neither toxic nor sensitive to degradation, OCQ can be a valuable tool for process automation and optimisation and to prolong thereby the productive time of these difficult to handle organisms. Due to its sensitivity, it can be probably used for control and maintenance of steady-state conditions e. g. as actuating variable for the dilution rate in perfusion processes or as feed control in fed batch processes, preliminary results are very promising (data not shown). Figure 5 illustrates that OCQ remains constant once steady state conditions are reached-in this case, the balance of CO_2_ to O_2_. The responsiveness of OCQ becomes clear in the first batch round, as shown in the graph. Due to a lower threshold, the stirring rate could not be decreased further to maintain pCO_2_ with the method mentioned above. Although effects on CO_2_ concentration are rather small, the OCQ illustrates this clearly by dropping below the expected steady state value, demonstrating the sensitivity of this method. Once the growth behaviour of a strain is known, as in the example above, OCQ and COQ can be used as simple triggers, or additional parameters to CER/OUR based control strategies in cases where these are not sensitive enough.

## Conclusions

The results of this study show that the importance of the proportion of O_2_ and CO_2_ for secondary metabolite production might have been underestimated or even neglected, at least for bacteria in the genus *Chondromyces*. Preliminary results with bacteria from the genus *Sorangium* show that these findings can be expanded to this bacterial group as well (data not shown) and it is possible that the productivity of other microorganisms will benefit also through tight control of the gaseous environment. With the method for CO_2_ and O_2_ maintenance described in this study, operators will have a powerful tool to investigate their influence with a relative simple instrumental setup. Furthermore, measurement of the off-gas allows by using the same principles used for controlling of the gaseous environment simple observation and control of bioprocesses with very few experiments and lack of detailed knowledge about media composition and intracellular biochemical processes by simply using very sensitive signals for respiratory activity.

## Methods

### Media and supplements

For cultivation of the *Chondromyces* strains, VY/2 media was used (5 g/l baker’s yeast, 1 g/l CaCl_2_ x 2H_2_O, 100 μl/l vitamin solution, 2 ml/l trace element solution), cultivation of *Saccharomyces cerevisiae* was carried out in M9-minimal media (6.78 g/l Na_2_HPO_4_ x 2H_2_O, 3 g/l KH_2_PO_4_, 1 g/l NH_2_Cl, 4 g/l Glucose, 2 ml/l of a 1 M MgSO_4_ solution, 100 μl/l of a 1 M CaCl_2_ solution, 2 ml/l trace element solution). Trace element solution (40 mg/l ZnCl_2_, 200 mg/l FeCl_3_ x 6H_2_O, 10 mg/l CuCl_2_ x 2H_2_O, 10 mg/l MnCl_2_ x 4H_2_O, 10 mg/l Na_2_B_4_O_7_ x 10H_2_O, 10 mg/l (NH_4_)6Mo_7_O_24_ x 4H_2_O). Vitamin solution 10 ml (4 mg Biotin, 64 mg Na-pantothenat, 64 mg nicotinamide, 64 mg pyridoxal-HCl, 14 mg p-amino benzoic acid, 14 mg cobalamine).

### Cultivation of microorganisms

Cultivation of *Chondromyces* strains was performed in repeated batch mode at 30 °C. Cells clumps were sedimented and depleted medium was replaced twice daily with fresh medium. A repetitive growth pattern, where biomass remains comparable in each cultivation cycle was reached after a few days. This result in a typical growth pattern with high oxygen requirement at the beginning of the cultivation cycle and decreasing requirement when nutrients are depleted as well as identical base, acid and oxygen consumption patterns indicating that comparable batch conditions are reached. Investigations were performed when this stage was reached. The testing conditions were maintained for one cultivation cycle before the measurement cycle. To maintain comparable conditions, the cultivation vessel was equipped in the same manner with a baffle and electrodes on the same position for every run. The filling volume of a 2 l vessel was 1.2 l the remaining volume was used as headspace. 200 ml cell clump volume remained in the reactor when media was exchanged. Cultivation of *Saccharomyces cerevisiae* was performed at 30 °C in minimal medium.

Two methods for maintenance of pO_2_ have been used and are indicated in the text. The first one achieves maintenance of pO_2_ by keeping the stirring rate at a constant level and changing the flow rate. The other method uses a constant flow rate and adapted stirrer speed for maintenance of pO_2_.

Relevant parameters were set under PI control using the following settings:

(3) Stirrer:min0,max800;P0.2,I0.2

(4) Flow:min0,max1.5;P0.7,I0.2

### Signal averaging

To smooth the signals the average of added signals from three minutes sampled every ten seconds was taken.

### Equipment and calibration

A Labfors 3 bioreactor microbial version (Infors Switzerland) was equipped with a 2 l vessel and Iris 5.2 for process control. O_2_ in the media was measured with a polarographic electrode (Mettler Toledo Switzerland) calibrated using two-point calibration with compressed air and nitrogen gas. pH was measured with an electrode (Mettler Toledo Switzerland) calibrated at pH 7.00 and 4.01. The off-gas analysis system consisted of a zirconium dioxide O_2_ sensor and an infrared CO_2_ sensor (Bluesens Germany). The sensors were calibrated for 30 min with ambient air before connecting them to the exhaust gas stream.

### HPLC/MS measurements

All measurements were performed on a Dionex Ultimate 3000 RSLC system using a Waters BEH C18, 100 x 2.1 mm, 1.7 μm dp column. Separation of 5 μl sample with Sulfadimethoxine as internal standard was achieved by a linear gradient with (A) H_2_O + 0.1% Formic acid (FA) to (B) Acetonitrile + 0.1% FA at a flow rate of 600 μl/min and 45 °C. The gradient was initiated by a 0.5 min isocratic step at 5% B, followed by an increase to 95% B in 18 min to end up with a 2 min step at 95% B. The LC flow was split to 75 μl/min before entering the maXis 4 G hr-ToF mass spectrometer (Bruker Daltonics, Bremen, Germany) using the standard ESI source. Mass spectra were acquired in centroid mode ranging from 150–2000 m/z at 2 Hz scan speed.

### Determination of differences in productivity

Metabolites were captured with XAD16 by incubating supernatant with resin for 1 h and subsequent extraction with 200 ml methanol. The extracts of the different cultivations were weighed and compared by adjusting concentration with methanol. Sulfadimethoxine was used as internal standard at a concentration of 6.75 mg/l. For comparison, twelve target compounds (TC) were chosen. The internal standard (IS) was to quantify the relative concentration of the twelve TCs in the six extracts by comparing the peak areas of the extracted ion chromatograms (EIC). To quantify the influence on overall productivity, the peak area of the TC is divided by the peak area of the IS. It is not possible to quantify the exact yield in mg/ml, but the overall differences in amount of the investigated substances in the six extracts can be calculated. The extract yield from the standard cultivation was used to calculate relative yield of the other extracts and TC/IS. An estimation of the influence of gaseous composition on overall productivity can then be made. For comparison of apicularens and crocapeptins, productivity concentration of the extract was adjusted and peak areas were compared.

### Determination of k_l_a

The dependency of k_l_a on stirrer speed and flow was determined with the dynamic method without oxygen consumer. The bioreactor was filled to the cultivation level with water and oxygen was stripped before every measurement cycle with nitrogen gas. For determination of the influence on k_l_a by bottom sparging a defined stirring speed and flow rate was adjusted for every cycle. For investigation of the influence of headspace aeration only a defined stirring rate was adjusted and a permanent exchange of headspace volume with air at 0.5 l/min was carried out. The maximum dissolved concentration of O_2_ at 30 °C was calculated to be 7.083 mg/l corresponding to the measured pO_2_ of 100%. The increase between 1-2.5 mg/l was linear at all conditions therefore k_l_a could be calculated by the following equation:

(5) $$k_{l}a=\frac{ln\left(\frac{7.083\frac{mg}{l}-1\frac{mg}{l}}{7.083\frac{mg}{l}-2.5\frac{mg}{l}}\right)}{t_{2.5}-t_{1.0}}$$

where t_1.0_ is the time point when 1 mg/l oxygen saturation is reached and t_2.5_ when 2.5 mg/l is reached

## Competing interests

The authors declare that they have no competing interests.

## Authors’ contributions

SH designed the experiments, carried out the studies, evaluated the data and wrote the manuscript. RM supervised the study and wrote the manuscript. All authors read and approved the final manuscript.
